# Long-term follow-up of cladribine treatment in hairy cell leukemia: 30-year experience in a multicentric Italian study

**DOI:** 10.1038/s41408-022-00702-9

**Published:** 2022-07-19

**Authors:** Livio Pagano, Marianna Criscuolo, Alessandro Broccoli, Alfonso Piciocchi, Marzia Varettoni, Eugenio Galli, Antonella Anastasia, Maria Cantonetti, Livio Trentin, Sofia Kovalchuk, Lorella Orsucci, Annamaria Frustaci, Angelica Spolzino, Stefano Volpetti, Ombretta Annibali, Sergio Storti, Caterina Stelitano, Francesco Marchesi, Massimo Offidani, Beatrice Casadei, Maria Elena Nizzoli, Maria Lucia De Luca, Luana Fianchi, Marina Motta, Luca Guarnera, Edoardo Simonetti, Andrea Visentin, Francesco Vassallo, Marina Deodato, Chiara Sarlo, Attilio Olivieri, Brunangelo Falini, Alessandro Pulsoni, Enrico Tiacci, Pier Luigi Zinzani

**Affiliations:** 1 Dipartimento di Diagnostica per Immagini, Radioterapia Oncologica ed Ematologia, Fondazione Policlinico Universitario A. Gemelli IRCCS, Roma, Italy; 2 Sezione di Ematologia, Dipartimento di Scienze Radiologiche ed Ematologiche, Università Cattolica del Sacro Cuore, Roma, Italy; 3 IRCCS Azienda Ospedaliero-Universitaria di Bologna Istituto di Ematologia “Seràgnoli”, Roma, Italy; 4 Dipartimento di Medicina Specialistica, Diagnostica e Sperimentale Università di Bologna, Bologna, Italy; 5 GIMEMA Foundation, Franco Mandelli Onlus, Rome, Italy; 6 Divisione di Ematologia, Fondazione IRCCS Policlinico San Matteo, Pavia, Italy; 7 Department of Hematology, ASST Spedali Civili, Brescia, Italy; 8 Dipartimento di Oncoematologia Policlinico Tor Vergata, Università Tor Vergata, Roma, Italy; 9 Division of Haematology and Clinical Immunology, University of Padova, Padova, Italy; 10 SOD C Ematologia, AOU Careggi, Firenze, Italy; 11 S.C. Ematologia, A.O.U. Città della Salute e della Scienza di Torino, Torino, Italy; 12 Divisione di Ematologia, ASST Grande Ospedale Metropolitano Niguarda, Milano, Italy; 13 Dipartimento di Medicina e Chirurgia, Università degli studi di Parma, Parma, Italy; 14 Oncoematologia, Istituto Oncologico Veneto IOV-IRCSS, Castelfranco Veneto, Italy; 15 Clinica Ematologia, Azienda Sanitaria Universitaria Integrata, Udine, Italy; 16 Hematology and Stem Cell Transplantation Unit, Campus Bio-Medico University, Roma, Italy; 17 UOC Oncoematologia Fondazione di Ricerca e Cura Giovanni Paolo II, Campobasso – Università Cattolica del Sacro Cuore, Campobasso, Italy; 18 Divisione di Ematologia, Azienda Ospedaliera “Bianchi Melacrino Morelli”, Reggio Calabria, Italy; 19 Haematology and Stem Cell Transplantation Unit, IRCCS Regina Elena National Cancer Institute, Roma, Italy; 20 Clinica di Ematologia, Azienda Ospedaliero-Universitaria Ospedali Riuniti di Ancona, Ancona, Italy; 21 Divisione di Ematologia, Department of Translational and Precision Medicine, Sapienza University of Rome, Rome, Italy; 22 Institute of Hematology and Center for Hemato-Oncology Research, Department of Medicine and Surgery, University and Hospital of Perugia, Perugia, Italy

**Keywords:** B-cell lymphoma, Chemotherapy

## Abstract

Hairy cell leukemia (HCL) is a rare lymphoproliferative disease with an excellent prognosis after treatment with cladribine (2CDA), although relapse may occur during follow-up. The aim of the study is to review the efficacy, safety, long-term remission rate, and overall survival (OS) in those patients who received 2CDA as first-line treatment. We retrospectively reviewed data of HCL patients treated with 2CDA between March 1991 and May 2019 at 18 Italian Hematological centers: 513 patients were evaluable for study purpose. The median age was 54 years (range 24–88) and ECOG was 0 in 84.9% of cases. A total of 330 (64.3%) patients received 2CDA intravenously and 183 (35.7%) subcutaneously. ORR was 91.8%: CR was obtained in 335 patients (65.3%), PR in 96 (18.7%), and hematological response in 40 (7.8%) patients; in 42 (8.2%) no response was observed. Hemoglobin value (*p* = 0.044), frequency of circulating hairy cells (*p* = 0.039), recovery of absolute neutrophil count (*p* = 0.006), and normalization of spleen (*p* ≤ 0.001) were associated with CR compared to PR in univariable analysis. At a median follow-up of 6.83 years (range 0.04–28.52), the median time to relapse was 12.2 years. A significant difference in duration of response was identified between patients that obtained a CR and PR (19.4 years versus 4.8 years, *p* < 0.0001). Non-hematological grade 3 or higher early toxicity was reported in 103 (20.1%) patients. Median OS was not reached: 95.3%, 92.4%, and 81.8% of patients were estimated to be alive at 5, 10, and 15 years, respectively. Forty-nine patients died (9.5%), following an infection in 14 cases (2.7%), natural causes in 14 (2.7%), cardiovascular events in 13 (2.5%), a second neoplasm in 6 (1.2%), and progression of HCL in 2 cases (0.4%). Following treatment of HCL with 2CDA, 80% of patients are estimated to be alive 15 years after diagnosis.

## Introduction

Hairy cell leukemia (HCL) is a rare lymphoproliferative disease with specific morphologic and molecular features. A complete diagnostic evaluation includes morphological recognition of typical hairy cells, characteristic immunophenotype with cell surface positivity of CD19, CD20, CD22, CD11c, CD25, CD103, and CD123, and the presence of BRAF-V600E mutation [1, 2]. HCL is characterized by an excellent prognosis: an extremely high rate of complete response (CR) has been reported after treatment with purine analogs, especially 2-chlorodeoxyadenosine (cladribine, 2CDA). The first report on the use of cladribine in HCL patients has been published in 1990: among 12 previously treated patients, 11 obtained a CR with a median duration of remission of 15.5 months [3]. Since then, several papers have focused on the response rate and duration of 2CDA in this setting of patients, even after intravenous or subcutaneous administration. One course of therapy can obtain a complete and durable response in 70–90% of patients [3–7], although relapses have been reported during long-term follow-up [7–10].

The main concern for the safety of patients is the occurrence of infections, both at the onset of disease and during bone marrow aplasia induced by chemotherapy: recovery of bone marrow function may require several weeks and sometimes a few months [11]. As largely addressed in the international guidelines for the treatment of HCL [12, 13], patients should be treated before peripheral blood counts deeply decline and/or an infection occurs. In case of clinically active infections and need for treatment, the choice of therapy should be carefully set on the single patients, while administrating broad-spectrum antibiotics and possibly antifungal therapy.

In the past 10 years, several new drugs have become available, such as BRAF inhibitors, MEK inhibitors, and anti-CD22 immunotoxin, with indications in specific clinical situations or beyond second-line treatment in clinical trials. Until these drugs will be licensed for HCL patients, purine analogs remain the standard of care for these patients. Considering the open issues in this setting, we decided to gather experiences of 18 Italian centers in the last 29 years: the primary endpoint of the study was an evaluation of the efficacy of single-agent 2CDA in the first line in routine practice. The secondary endpoint of the study was an evaluation of the safety of treatment, according to early adverse events and long-term responses.

## Materials and methods

We retrospectively reviewed data of patients with HCL treated between March 1991 and May 2019, at 18 Italian Hematological centers. We recruited all patients treated with 2CDA as first-line monotherapy, to evaluate the efficacy, safety, and long-term remission rate of 2CDA.

Demographic and hematological data were registered in an electronic data form. In particular, all participating centers collected information about age, sex, performance status according to the ECOG scale, concomitant chronic diseases, complete blood counts, and bone marrow evaluation at the onset of disease and before treatment. Schedule of chemotherapy administration, adverse events during cytopenia (allergic reactions, thrombotic and hemorrhagic complications), location and severity of infections, duration of cytopenia and bone marrow recovery, depth of remission, characteristic and treatment of relapse, long-term adverse events, and survival were also recorded. Indications for starting treatment, response evaluation, and relapse criteria were defined as per recent consensus guidelines [12]. Response criteria were defined as reported in Table 1. In patients achieving CR or partial response (PR), loss of response was defined as worsening of peripheral blood count under the thresholds reported for CR and PR. As patients may show morphological evidence of disease without experiencing cytopenias [14], indications for retreatment comprise a re-occurrence of disease-related symptoms together with 25% increase in splenomegaly and 25% reduction in peripheral blood counts, compared to levels obtained at the time of response. Relapse-free survival (RFS) was defined as the time from response achievement until the occurrence of the need for retreatment or death from HCL-related causes or the date of the last follow-up. Overall survival (OS) was defined as the time from diagnosis to death from any cause or the date of the last follow-up.

**Table 1:** Definition of response criteria.

CR	Recovery of hemoglobin above 11 g/dl, platelets above 100 × 10^9^/l, ANC above 1.5 × 10^9^/l
	Normal spleen diameter by physical exam
	No evidence of disease both in peripheral blood and bone marrow by non-immunological stains
PR	Recovery of peripheral blood count as for CR
	At least 50% reduction of splenomegaly
	At least 50% reduction of bone marrow infiltration by morphology
HR	Improvement by 50% of peripheral blood counts
NR	No improvement in peripheral blood count
	No reduction of splenomegaly
	No reduction of bone marrow infiltration

Our research has been conducted following Helsinki Declaration principles and approved by the local ethical committees. Informed consent was obtained from all subjects.

### Statistical analysis

A descriptive analysis of the demographic and clinical characteristics of the patients was performed, including median and range for continuous variables, and absolute and relative frequencies for categorical variables. Non-parametric tests were used to evaluate differences among groups (Fisher exact test and Wilcoxon test for categorical and continuous variables, respectively). Survival curves (OS and RFS) were estimated according to the Kaplan–Meier product-limit method and were tested for significant differences using the log-rank test in univariate analysis and using the Cox regression model in multivariate analysis. In all analyses, 95% confidence intervals (CI) were reported for the main summary statistics and all statistical comparisons were based on two-tailed tests accepting *p* ≤ 0.05 as statistically significant. All analyses were performed R software (R: A language and environment for statistical computing. R Foundation for Statistical Computing, Vienna, Austria).

## Results

### Clinical characteristics

We collected 557 patients from 18 centers, among which 513 were evaluable for the study purpose and characterized in Table 2. Forty-four patients (7.9%) were excluded because of suspected diagnosis of HCL variant (17 cases), which generally have a reduced rate of response compared with classic HCL, concomitant treatment with Rituximab (15 cases), or incomplete data (12 cases).

**Table 2:** Characteristics of patients.

	*n*	Range, %
Sex		
M	420	81.9
F	93	18.1
Age (median)	54	24–88
Complete blood count		
Hemoglobin (g/dl, median)	12.2	3.4–16.8
White blood cell count (×10^9^/l, median)	2.8	0.6–77.88
Absolute neutrophil count (×10^9^/l, median)	0.864	0.25–9.512
Absolute lymphocyte count (×10^9^/l, median)	1.639	0.15–15.042
Circulating hairy cells	138	26.8
Platelet count (×10^9^/l, median)	78	1–397
Bone marrow data		
Bone marrow total cellularity (%)	55	8–100
Bone marrow leukemic cellularity (%)	80	8–100
BRAF-V600E mutation (available in 136 patients)	136	100
Onset characteristics		
Splenomegaly	241	46.9
Hepatomegaly	42	8.2
Lymphadenomegaly	30	5.8
ECOG		
0	436	84.9
1	71	13.9
2	6	1.2
Comorbidity		
Infection	37	7.2
Cardiovascular disease	29	5.6
Previous cancer	27	5.2
Diabetes	27	5.2
Hepatic disease	18	3.5
CODP	16	3.1
Chronic kidney disease	3	0.6

Male/female ratio was 4.5 with a median age of 54 years (range 24–88); more than half of patients (298, 58%) were diagnosed in the 2010–2019 years. BRAF-V600E mutation result was only available for 136 (26.4%) patients and positive in all cases. Splenomegaly was detected in 241 (46.9%) patients, with a median diameter of 18 cm (range 15–30) in the 230 patients with this radiological information available. At the onset, 436 patients (84.9%) were in good clinical condition with ECOG 0, while 71 (13.9%) and 6 (1.2%) had an ECOG 1 and 2, respectively. We recorded a low comorbidity rate in this population. Thirty-seven (7.2%) patients presented with an infection at the onset of disease, that was pneumonia in 24 cases (64.9%), fever of unknown origin in 7 (20%), skin and abdominal abscess in 2 (5%), and 4 (10%) cases respectively.

### Treatment

All patients received 2CDA, after a median time from diagnosis of 1.2 months (range 0.1–261.8) (Table 3), administered intravenously in 330 (64.3%) patients and subcutaneously in 183 (35.7%) patients. Among patients with intravenous 2CDA, 250 (75.8%) received a daily infusion for 5–7 consecutive days and 80 (24.2%) a weekly infusion for 5–7 consecutive weeks.

**Table 3:** Treatment of patients and response to treatment.

	*n*	Range, %
Route of administration		
iv	330	64.3
sc	183	35.7
Time to treatment (months, median)	1.2	0.1–261.8
Blood count recovery		
White blood cell count (day, median)	30	4–302
Platelet count (day, median)	22	6–300
Hemoglobin (day, median)	50	7–182
Time to evaluation of response (months, median)	3.3	0.3–9.8
Response		
CR	335	65.3
PR	96	18.7
HR	40	7.8
NR	42	8.2
Second course	25	
CR	13	52
PR	5	20
HR	2	8
NR	5	20
Status		
Alive	464	90.5
Dead	49	9.5
OS at 25 years, %	66	50.7–85.6

*CR* complete response, *PR* partial response, *HR* hematological response, *NR* no response.

### Adverse events

Overall, adverse events were reported in 175 patients (34.1%) (Table 4). Allergic reactions were reported in 48 (9.2%) patients, only 1 of which was recorded as grade 3. Hemorrhagic events were reported in 3 (0.6%) patients: 2 cases of low-grade skin bleeding and 1 case of grade 3 gastrointestinal bleeding; 3 (0.6%) cases of thrombosis, deep vein of lower limbs (2), and peri-catheter upper limb (1), were also reported. Other adverse events (5 hepatic, 1 gastrointestinal, and 1 cardiovascular; 1.4%) were reported: 4 were grade 3 (3 hepatic and 1 gastrointestinal) and 2 were grade 4 (1 hepatic and 1 cardiovascular). Infection was reported in 141 (27.7%) cases: 97 (19.2%) were treated as inpatients due to grade 3 or higher severity of the event, whereas 44 were treated as outpatients due to grade 2 or lower severity. Most infections were recorded as fever of unknown origin (98, 69.5%): these events are more likely the result of cytokine release rather than true infection. Pneumonia was detected in 23 (15.6%) patients: in 5 cases it was clinically documented only, while in 18 cases it was attributable to bacteria (8, 44.5%), fungi (6, 33.4%), or viruses (4, 22.1%). Other sites of infections were bloodstream (9, 6.8%), upper respiratory tract (4, 2.7%), skin (4, 2.7%), and lower urinary tract (3, 2.7%). Overall, 6 patients died of infectious complications: none of them presented an uncontrolled infection before treatment. The 6 fatal infective events included invasive aspergillosis, bacterial pneumonia, and bacterial sepsis (2 cases each).

**Table 4:** Adverse events and grading.

		Grade 1	Grade 2	Grade 3	Grade 4	Grade 5
Infections	141	44		61	30	6
Inpatient	97					
FUO	98					
Lung	23					
BSI	9					
Upper respiratory tract	4					
Skin	4					
Lower urinary tract	3					
Allergy	48					
Urticaria	47	47				
Dress syndrome	1			1		
Hemorrhage	3					
Skin	2		2			
GI tract	1			1		
Thrombosis	3					
Limbs	3		3			
Other	7					
Hepatic	5		1	3	1	
Cardiovascular	1				1	
GI tract	1			1		

Hematological toxicities are detailed in Table 5. Briefly, among 111 patients with ANC > 1.5 × 10^9^/l, grade 2 and above neutropenia occurred in 29 patients (26%), while among the 295 patients with grade 2 or 3 neutropenia worsening to grade 3 and 4 occurred in 128 cases (43%). In 49 patients with normal platelet count, grade 1–2 thrombocytopenia occurred in 14 cases (29%), while among the 464 thrombocytopenic patients, worsening to grade 2 and above occurred in 108 cases (23%). In 357 patients with hemoglobin level above 11 g/dl, grade 1 to 3 anemia occurred in 102 cases (29%; grade 2 in 55 cases, 15%), while among 157 patients with anemia grade 1 or above worsening to grade 2 or above occurred in 48 cases (31%).

**Table 5:** Hematological toxicities and grading.

Level	*n*	Grade 1	Grade 2	Grade 3	Grade 4
ANC >1.5 × 10^9^/l	111		3	13	13
ANC grade 2	119			21	34
ANC grade 3	176				73
ANC grade 4	107				
plt >150 × 10^9^/l	49	12	2		
plt grade 1	235		45	9	5
plt grade 2	129			26	8
plt grade 3	80				15
plt grade 4	20				
Hb >11 g/dl	357	33	55	14	
Hb grade 1	37		10	9	
Hb grade 2	62			27	
Hb grade 3	50				2
Hb grade 4	7				

Transfusions of red blood cells (median 3 units; range: 1–27) and platelets (median 2 units; range: 1–9) were required in 98 (19%) and 17 (3%) of all 513 treated patients, respectively. The timing of cytopenia recovery was as follows (Table 2). In 284 neutropenic patients, recovery above 1.5 × 10^9^/l of ANC was reported in 169 (59.5%) patients after a median of 30 days (range 4–302). Recovery above 100 × 10^9^/l of platelet count was reported in 214 (59%) of 363 thrombocytopenic patients after a median of 22 days (range 6–300), while recovery above 11 g/dl of hemoglobin value was reported in 81 (68.1%) of 119 patients with anemia after a median of 50 days (range 7–182).

### Response to the treatment

**Fig. 1:**
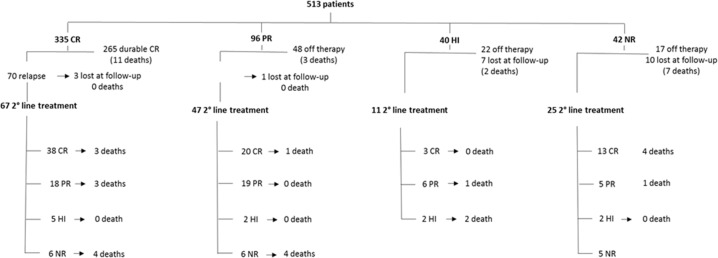
Response to first-line and salvage therapy. The diagram shows the deep of response and the outcome after first-line therapy, the number of patients who underwent a second line teatment, and the deep of response and outcome of second-line therapy. CR complete response, PR partial response, HI hematological improvement, NR no response.

Response was evaluated after a median time of 3.3 months (range 0.3–9.8). Overall response rate (ORR) was 91.8%: CR was obtained in 335 (65.3%) patients, PR in 96 (18.7%) and hematological response (HR) in 40 (7.8%); 42 (8.2%) patients did not show any response (no response) (Fig. 1). Characteristics associated in multivariate analysis with CR compared to PR were higher hemoglobin value (OR: 1.12, 95% CI: 1.02–1.22, *p* = 0.021) and lower number of circulating hairy cells (OR: 3.72, 95% CI: 1.35–13.2, *p* = 0.020 (Table 6). No other differences between complete versus partial responders were identified, according to sex, age, calendar decade of treatment, route of administration, baseline values of white blood cells, neutrophils, and platelets, comorbidities at onset, time from diagnosis to treatment start, percentage of leukemic infiltration and bone marrow Hairy Cell Index (total bone marrow cellularity multiplied by leukemic cellularity).

**Table 6:** Univariate analysis for characteristics associated with CR compared to PR.

	Univariate analysis			Multivariate analysis		
	OR	95% CI	*p* value	OR	95% CI	*p* value
Age	0.99	0.97, 1.01	0.26			
Year of diagnosis						
1990–2009	–	–				
2010–2019	1.11	0.70, 1.74	0.66			
Sex						
M	–	–				
F	0.72	0.42, 1.27	0.25			
ECOG						
0	–		–			
1–2	2.13	0.99, 5.30	0.074			
Hb (g/dl)	1.09	0.99, 1.19	0.072	1.12	1.02, 1.22	**0.021**
Plts	1	1.00, 1.00	0.32			
Wbc	1	1.00, 1.00	0.36			
Circulating hairy cells						
Yes	–	–				
No	3.66	1.34, 12.9	**0.021**	3.72	1.35, 13.2	**0.02**
Time to treatment	1	0.99, 1.01	0.5			
Route of administration						
IV	–	–				
SC	1.2	0.74, 1.97	0.47			
CODP						
No vs Yes	0.26	0.01, 1.31	0.19			
Diabetes						
No vs Yes	0.65	0.18, 1.75	0.43			
Hepatic disease						
No vs Yes	2.24	0.66, 6.90	0.17			
Cardiovascular disease						
No vs Yes	1.21	0.43, 3.02	0.69			
Previous cancer						
No vs Yes	0.75	0.21, 2.07	0.61			
Splenomegaly at onset						
No vs Yes	0.93	0.58, 1.48	0.76			
Lymphoadenomegaly at onset						
No vs Yes	2.11	0.86, 4.93	0.089			
Hepatomegaly at onset						
No vs Yes	1.46	0.66, 3.01	0.32			

*OR* odds ratio, *CI* confidence interval.

Bold values refer to statistically significant results.

Among 42 non-responder patients, 25 (4.9%) underwent a second course of treatment: 13 obtained a CR (7 with rituximab and 6 with 2CDA), 5 a PR (2 with 2CDA, 2 with pentostatin, 1 with interferon-α, and 1 with rituximab) and 2 an HR (2 cases with interferon-α). Five patients did not improve after receiving 2CDA (2 cases), rituximab (2 cases), or interferon-α (1 case). Of the remaining 17 non-responder patients, 10 were lost to follow-up a few weeks after bone marrow re-evaluation and 7 died soon after first treatment. Overall, 11 and 4 patients deceased of natural causes and infections, respectively, 7 before and 8 after second-line therapy (Fig. 1).

**Fig. 2:**
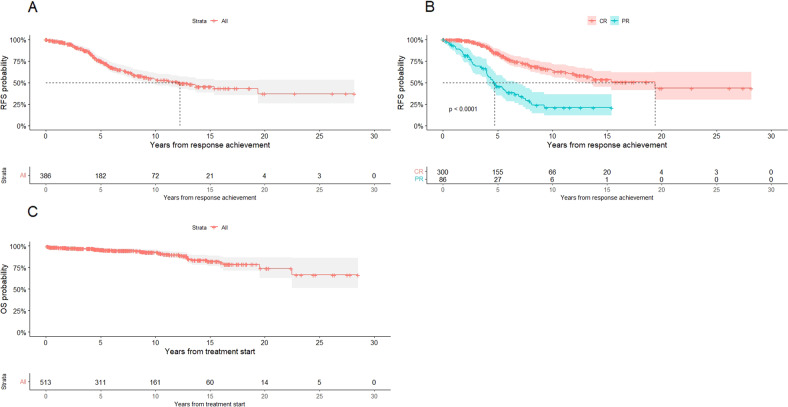
Survival curves of the study population. **A** Global RFS of the study population. **B** RFS of the study population according to response. **C** OS of the study population.

At a median follow-up of 6.83 years (range (0.04–28.52) in the 431 patients achieving CR or PR, the median time to relapse was 12.2 years: particularly, 75%, 53.6%, and 45.4% of patients were expected to be free from relapse at 5, 10, and 15 years, respectively (Fig. 2A). A statistically significant difference in duration of response was identified between patients that obtained a CR compared to patients in PR (19.4 years versus 4.8 years, *p* < 0.0001) (Fig. 2B). No other differences in RFS were identified, according to the route of administration, detection of BRAF-V600E mutation, calendar decade of treatment, age at treatment start, sex, baseline values of white blood cells, neutrophils, platelets and hemoglobin, and infection at onset, percentage of leukemic infiltration and bone marrow Hairy Cell Index. No differences in depth and duration of response, RFS, infections, bone marrow recovery, and survival were reported among 183 patients receiving subcutaneous administration of 2CDA, compared to intravenous administration.

Of all 431 responders, 118 (27.3%) patients relapsed, including 70 of the 335 with a previous CR (20.9%) and 48 of the 96 with a previous PR (50%). Among these 118 relapses, 4 patients were lost to follow-up whereas 114 patients underwent a second-line therapy (Fig. 1) after a median of 53.2 months from response (range 7.5–211.5): 85 (74.5%) patients were retreated with 2CDA, alone (64 cases) or associated with rituximab (21 cases); 12 patients (10.5%) with pentostatin, alone (8 cases) or associated with rituximab (4 cases); 8 patients (7%) with interferon α, 7 patients (6.1%) with rituximab, 1 patient (0.9%) with vemurafenib and zanubrutinib each. ORR2 was 89.4%: 58 patients (50.9%) obtained a CR (42 after 2CDA), 37 patients (32.4%) a PR (32 after 2CDA), 7 patients (6.1%) an HR (4 after 2CDA) and 12 patients (10.5%) had no response (6 after 2CDA) (Fig. 2). Regarding the 40 patients obtaining only HR after first-line cladribine (Fig. 1), 11 patients underwent a second-line treatment after a median time of 4.3 years (range 0.9–17.7 years): 9 (81.8%) were retreated with cladribine and 2 (18.2%) were treated with pentostatin. ORR was 81.8%: 3 and 6 patients obtained CR and PR, respectively. Moreover, 22 patients did not receive any other treatment and 7 were lost to follow-up.

Median OS was not reached: particularly, 95.3%, 92.4%, and 81.8% of patients were estimated to be alive at 5, 10, and 15 years, respectively (Fig. 2C). Overall, 49 patients (9.5%) deceased: causes of death were infection in 14 cases (2.7%), natural causes in 14 (2.7%), cardiovascular events not related to HCL or treatment in 13 (2.5%), a second neoplasm in 6 (1.2%) and progression of HCL in 2 cases only (0.4%).

## Discussion

We reported on a population of 513 patients affected by HCL and treated with 2CDA alone in the first line in the last 29 years. To our knowledge, this is the largest real-life study of HCL patients treated with 2CDA in the first line, despite some limitations.

The retrospective nature of data collection and the different local practices may have reduced an exhaustive evaluation of response. Second, different staining procedures have been used during the extent of the study period, which may have caused a non-uniform evaluation of bone marrow biopsy and affected the correct classification of response and ultimately CR rate. Third, another limitation of this study is the lack of minimal residual disease analysis—which has recently been described to be an important prognostic marker. Lastly, some patients may have been lost to follow-up considering the median time of study observation. However, this study has allowed us to collect several useful information on this patient setting regarding the efficacy and safety of the treatment received. First-line treatment schedule has not really changed, except for the introduction of subcutaneous administration. On the other hand, we showed that the survival rate has increased over the years, likely because of better management of adverse events and correct timing of response evaluation.

The discovery of BRAF mutation in 2011 [15] has improved the differentiation of HCL from other overlapping lymphoproliferative diseases. Although this molecular analysis has been widely performed only in the last years, other clinical and flow cytometry characteristics are helpful to discriminate HCL from variants and lymphomas. In fact, our population is quite uniform, considering the low percentage of leukocytosis (<5%) and the ubiquitarian presence of surface markers such as CD25, CD123, and CD11c. The comorbidity rate was low, probably due to the median age of 54 years and less than 15% of patients aged over 70 years. Few patients presented with cardiovascular disease and obstructive pulmonary disease, which are the most frequent comorbidities reported in the general population; a previous neoplasm was reported in 5.3% of cases.

In our population, 2CDA was administered intravenously in 330 cases, as a daily continuous infusion for 5–7 days or a weekly infusion for 5–7 weeks, and subcutaneously in 183 patients. No differences in terms of quality, duration of response, and complications were reported, as previously published [5, 16–18].

Overall, one-third of patients experienced an all-grade adverse event. Fever of unidentified origin was reported in 98/141 infective events (69.5%) and was more likely the result of cytokine release rather than true infection. Of note, 7.2% of patients presented an infectious complication at the onset of malignancy, mostly pneumonia. Conversely, acute mortality due to infections was low; in fact, we observed only 6 cases of deadly infectious complications: none of the patients presented an infection at the onset of the disease. In 1998, Cheson et al. [19] reported a 31% rate of infections among 895 patients, with 3.3% of deaths due to infections. In 2011, Kraut [11] reported the data in aggregate of 5 large studies: among 1444 patients treated with 2CDA, 32% of infections including neutropenic fever were reported, and 0.4% deaths due to infections. Recently, a French group [20] reported 15% of infections among 279 patients during follow-up, including 3 cases of invasive aspergillosis. Comparing our data with these studies, we observed a marked reduction in both infections and infection-related death. Probably, this is due to the improvement of anti-infective supportive therapy introduced over the years, and a better understanding of malignancy. We do not report any infection during long-term follow-up among patients who responded to treatment.

According to a clinical practice approach, in 1998 the largest study on HCL patients reported a CR rate of 50% and a PR rate of 37% for 2CDA monotherapy, although only 42% of patients were previously untreated [19]. In our study, the CR rate is only 64%, which is quite lower than the 80–90% CR rate commonly reported in the literature [3, 7, 9, 10, 20]. This could be ascribed to a median time to evaluation of the response of only 3.3 months, which is shorter than 5–6 months considered necessary for eliminating bone marrow infiltration [12]. The low proliferative index, typical of hairy cells, may produce persistence of bone marrow infiltration not leading to relapse [14], but related to slow clearance of neoplastic cells. Among patients evaluated for a response after a minimum of 4 months, the CR rate was 70% and the ORR was 89%, closer to data reported in the literature. Similarly, a prospective study on first-line subcutaneous 2CDA reported an ORR of 88% with only 48.6% of CR, assessed early after 71 days from the last administration of treatment [18].

Although the percentage of CR reported in this study may be low, for the reasons listed above, it should be noted, however, that the survival curves showed an extremely satisfactory percentage of long-term survival, demonstrating the effectiveness of the treatment.

Another point is the recent increase of immune-histochemical stains to assess response to therapy. This method has a higher resolution compared with hematoxylin and eosin stain, which is the morphological criterion historically used to define CR. This may have contributed to a lower CR rate, and makes our results not perfectly comparable to previous reports.

In a previous study on 233 patients treated with purine analogs, Hb level <10 g/dl and platelet count <100 × 10^9^/l at diagnosis led to inferior RFS both in patients with CR and PR [7]. In our population, peripheral blood characteristics at presentation as a slightly higher hemoglobin value and lower number of circulating hairy cells were associated with the best response in multivariate analysis (CR compared to PR). Moreover, a statistically significant different duration of response was reported among patients which obtained CR compared to those obtaining PR, which is in line with recent literature [7, 8, 20].

Median RFS was 12.2 years in our population, with 75% and 45.4% of patients expected to be free from relapse at 5 and 15 years, respectively. One hundred fourteen relapsed patients (22%) received a second-line therapy: 84 patients were treated with 2CDA and obtained a 50% CR2 and 38.1% PR2 (ORR2 88.1%), which is still a considerable result. Huynh et al. reported a relapse rate of 19% at 48 months among 341 responding patients after 2CDA in the first line: after the second course of 2CDA was given in 53 patients, 33 (62%) achieved a CR2 and 14 (26%) a PR2 with an ORR2 88% [21]. Paillassa et al. reported a median RFS of 163 months in a cohort of 159 patients treated with the first-line 2CDA, and median OS was not reached [20]. Similarly, in our population median OS was not reached: more than 80% of patients were estimated to be alive up to 15 years after diagnosis.

Lethal complications occurred in 9.5% of patients in our study population, with few patients dying of HCL-related events. In the study of Cheson et al. mortality rate was 13.5% (125/928 treated patients), mostly attributed to the progression of disease and infectious complications [19]. Else et al. in 2009 reported an overall mortality rate of 19% among 233 patients treated with purine analogs, which was not significantly different from the general population matched for age and sex [7]. These results demonstrate that despite the use of a single-agent, non-targeted treatment, improvement of supportive therapy has reduced early death and increased long-term remission rate and survival. Since the report of an estimated 5-year survival rate of 85% by Cheson et al. in 1998 [19], the prognosis of these patients has improved: we reported an estimated 5-year survival rate of 95.3%, with a lower rate of HCL-related deaths.

2CDA is a safe and effective therapy both in the first line and in the salvage setting. Although the use of purine analogs has changed the natural history of HCL, infective complications are still a concern, especially at the onset of disease and at the occurrence of relapse. On the other hand, the mortality rate has constantly lowered during the last years and 80% of patients were estimated to be alive, and largely without clinical signs of disease, up to 15 years after diagnosis. Nevertheless, advances can still be made to optimize treatment of these patients. Recently, the concomitant or subsequent use of antiCD20 monoclonal antibody together with 2CDA has suggested increased CR rates compared to 2CDA alone [22]. Moreover, several targeted drugs have shown considerable results among relapsed patients enrolled in clinical trials [23–29]. These data will probably change the therapeutic approach to HCL: in the next future, incorporation of newer agents will be useful to further improve the long-term outcome of these patients. At present, 2CDA still remains the backbone of treatment in this setting of patients.

## Data Availability

The datasets used and/or analyzed during the current study are not publicly available due to individual privacy reason, but are available from the corresponding author on reasonable request.
